# A Planning Framework Based on Semantic Segmentation and Flipper Motions for Articulated Tracked Robot in Obstacle-Crossing Terrain

**DOI:** 10.3390/biomimetics10090627

**Published:** 2025-09-17

**Authors:** Pu Zhang, Junhang Liu, Yongling Fu, Jian Sun

**Affiliations:** School of Mechanical Engineering and Automation, Beihang University, Beijing 100191, China; zpungry@buaa.edu.cn (P.Z.); buaa706ljh@buaa.edu.cn (J.L.); fuyongling@buaa.edu.cn (Y.F.)

**Keywords:** articulated tracked robots, obstacle-crossing terrain, DeepLab V3+, map semantic segmentation, RRT

## Abstract

Articulated tracked robots (ATRs) equipped with dual active flippers are widely used due to their ability to climb over complex obstacles like animals with legs. This paper presents a novel planning framework designed to empower ATRs with the capability of autonomously generating global paths that integrate obstacle-crossing maneuvers in complex terrains. This advancement effectively mitigates the issue of excessive dependence on remote human control, thereby enhancing the operational efficiency and adaptability of ATRs in challenging environments. The framework consists of three core components. First, a lightweight DeepLab V3+ architecture augmented with an edge-aware module is used for real-time semantic segmentation of elevation maps. Second, a simplified model of the robot-terrain contact is constructed to rapidly calculate the robot’s pose at map sampling points through contact point traversal. Finally, based on rapidly-exploring random trees, the cost of flipper motion smoothness is incorporated into the search process, achieving collaborative planning of passable paths and flipper maneuvers in obstacle-crossing scenarios. The framework was tested on our Crawler robot, which can quickly and accurately identify flat areas, obstacle-crossing areas, and impassable areas, avoiding redundant planning in non-obstacle areas. Compared to manually operated remote control, the planned path demonstrated shorter travel time, better stability, and lower flipper energy expenditure. This framework offers substantial practical value for autonomous navigation in demanding environments.

## 1. Introduction

**Figure 1:**
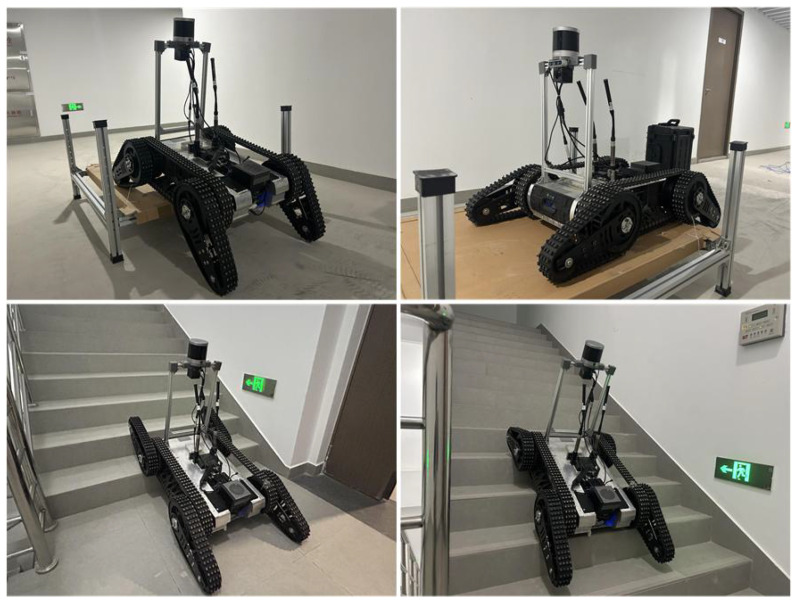
Climber robot moving in obstacle-crossing scenarios.

From emergency disaster relief to environmental exploration and infrastructure maintenance [1,2], mobile robots are transforming various industries. Complex terrain poses challenges for the movement of mobile robots. Through thousands of years of evolution, creatures in nature have developed the ability to effortlessly climb obstacles using their flexible limbs. Humans seek inspiration from them, leading to the development of snake-like robots, legged robots, and other such designs.Articulated tracked robots equipped with dual active flippers are extensively deployed for their superior obstacle negotiation performance. The wider track contact area provides excellent stability. Meanwhile, compared to traditional crawler robots, they are as flexible as legged animals and can adapt to different complex terrains by changing the angle of their flippers, thus easily crossing obstacles. Our Climber robot is designed on the basis of this principle, shown in Figure 1.

Traditional systems rely on remote teleoperation, which requires simultaneous manual control of both locomotion and flipper angles, which imposes a significant burden on the operator. With advancements in simultaneous localization and mapping (SLAM) technology and increased computational power, 2D indoor ground mobile robot navigation has become relatively mature. By improving the robustness and efficiency of existing 2D autonomous navigation solutions [3,4,5], autonomous navigation has been widely adopted in various fields [6,7]. However, navigating articulated tracked robots in obstacle-crossing terrain remains challenging, as their trajectories extend beyond the planar workspace. There exist highly variable obstacle-crossing maneuvers and, at the same time, they are different from the 3D motion planning of UAVs because the robots always have to make contact with the ground to move. Therefore, autonomously planning a stable and passable path that includes active front and rear flipper movements is a significant challenge for robots to navigate complex obstacle-crossing scenarios.

Efficient environment representation is a prerequisite for complex scene navigation. Traditional methods rely on geometric representations such as 2.5D height maps [8,9], point clouds [10], or truncated signed distance fields (TSDF), which encode topological and geometric features but lack semantic information needed for advanced tasks [11]. Vision-based methods are widely adopted to embed semantic understanding [12]. For example, Tateno et al. [13] leveraged CNN-based depth estimation integrated with monocular SLAM to achieve semantic mapping.However, integrating these methods with LiDAR and IMU SLAM systems requires additional visual sensors. Wellhausen et al. [14] trained CNNs to infer foothold semantics for legged robots using elevation maps.

Several studies have explored Articulated Tracked Robot navigation in complex terrains, emphasizing flipper angle adaptation. Discrete flipper states effectively address terrain-specific configurations. A D-Lite-based planner [15] selects the optimal angle of the flipper from four predefined poses based on terrain data; however, it treats position and flipper planning as separate processes, leading to suboptimal integration. These methods often overlook motion smoothness, leading to inefficiencies and high energy consumption as a result of frequent state switching. A simplified skeleton model [16,17,18] enables continuous motion generation by searching a degenerate configuration space but prioritizes local transitions over global efficiency.

More recently, a planar RTI model [19] has been proposed to describe robot-terrain interactions. It formulates the hybrid terrain optimization (HTO) problem as a multi-objective NLP solved in real time to switch between travel and traversal modes. However, its lack of global path planning hinders path and motion planning coordination. Neural networks [20] and reinforcement learning [21,22] have been applied to flipper planning but remain limited to specific terrains, therefore, the objective of this study is to develop a universal planning methodology that is adaptable to diverse terrains. For ATRs, commonly deployed in search and rescue missions, efficient obstacle-crossing requires coordinated path and flipper motion planning. However, existing methods struggle to integrate both seamlessly, highlighting the need for improved autonomous navigation in complex environments.

**Figure 2:**
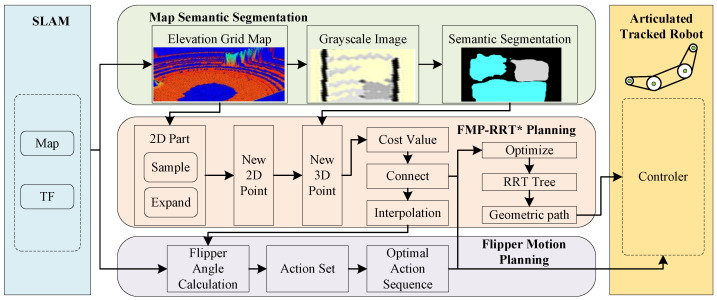
The planning framework based on semantic segmentation and flipper motions for articulated tracked robots in obstacle-crossing terrain.

Inspired by animals’ agile movement in complex environments, we aim for robots to possess biological-like semantic understanding of maps, enabling them to plan their own limb movements and feasible global paths.Therefore, we propose a planning framework for Articulated Tracked Robots in obstacle-crossing terrains (Figure 2), which contains three core components: (1) a lightweight DeepLab V3+ network with an added edge awareness module for map semantic segmentation; (2) a contact-point traversal-based computation of flipper motions; and (3) a RRT * algorithm based on the computation of flipper motions to generate global path, which takes into account the obstacle-crossing difficulty cost and the flipper traveling stability cost. The framework realizes the collaborative planning of path and flipper actions in obstacle-crossing terrains, reduces the operator’s manipulation burden, and produces smooth and efficient motions.

The structure of this paper is as follows: in Section 1, we describe the background of this study, related work, and the structure of the article. In Section 2 semantic sgmentation of the environment perceived by the robot is discussed, with the main algorithmic framework based on an improved DeepLab V3+ network; Section 3 describes the method for calculating the robot’s arm angle; Section 4 introduces the global path optimization method based on RRT*; Section 5 describes the implementation of the navigation framework on Climber; and the conclusions are presented in Section 6.

## 2. Map Semantic Segmentation

**Figure 3:**
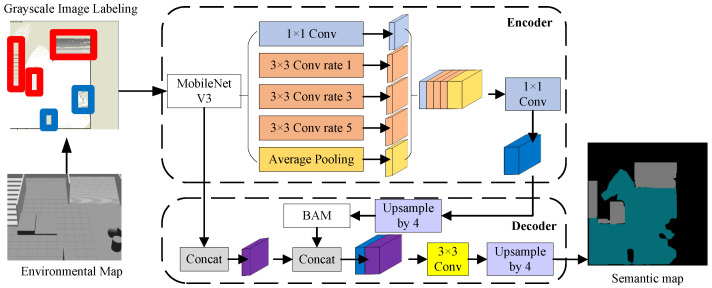
The MB-DeepLab V3+ architecture incorporates a MobileNet V3 encoder and BAM decoder modules within the DeepLab V3+ framework.

**Figure 4:**
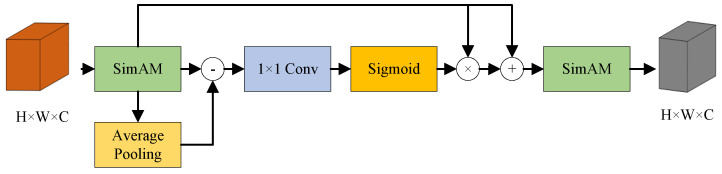
Boundary Aware Module(BAM).

We trained a DeepLab V3+ network that combines MobileNet V3 and the boundary aware module method (MB-DeepLab V3+) for map semantic segmentation, divide the map into flat areas, obstacle-crossing areas, and impassable areas, and store the map semantics in τ for use in subsequent calculations. The framework is shown in Figure 3. We converted the point cloud map to elevation raster map data as input, a region of the elevation raster map centered on the robot was extracted, and mapped the height values in the area to a grayscale image of 224 × 224 size. Participate in training by annotating grayscale images, red indicates the obstacle-crossing area, and blue indicates the impassable area.Then several improvements are made based on DeepLab V3+ [23], a widely used semantic segmentation framework: (1) the encoder adopts the lightweight MobileNet V3 [24] as the feature extraction network and changes it to a single channel input; (2) Since the output feature map size of MobileNet V3 is 7 × 7, we changed the dilation coefficients of the dilated convolution in ASPP to 1, 3, and 5. This ensures that the context information of different scales is covered, while avoiding insufficient information due to the receptive field exceeding the boundary. (3) The part of the CBM module in the B2Cnet network [25] that performs edge feature enhancement of the feature map is extracted and added to the decoder as an edge-aware module boundary-aware module (BAM), shown in Figure 4, which consists of the lightweight attention mechanism SimAM, the average pooling layer, 1 × 1 convolution and Sigmod activation function.

## 3. Calculation of Flipper Posture

### 3.1. Simplified Modeling of the Robot

We use the Climber robot (Figure 1), which is equipped with front and rear flippers on a standard tracked chassis; the front two flippers are driven by one drive motor to realize simultaneous up and down pitch, and the same principle of the rear two flippers. The robot is symmetric in structure on both sides.

**Figure 5:**
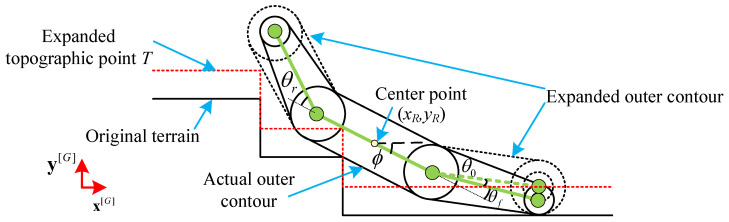
Simplified model of articulated tracked robot contact with terrain.

In the lateral projection, since the diameter of the small wheel of the flipper is different from that of the big wheel, making the definition of the angle of the flipper complicated, the small wheel is conceptually expanded to match the diameter of the large wheel (Figure 5). The dotted line is the outline of the expanded flipper. When calculating the angle of the front and rear flippers, the angular offset $\theta_{0}$ introduced by the virtual expansion of the flipper wheels is considered. At this point, the contour of the ground can be extended outward along the normal direction of the contour, with the extension distance being the radius of the track wheels. The robot is then simplified into a three-link structure and a thick-free robot model is obtained that is convenient for calculation (the green line segment in Figure 5). After expansion, complex geometric calculations in the literature [19] can be avoided, improving the efficiency of subsequent calculations.

The terrain beneath the robot is represented by a discrete set of points. The local terrain can be sensed by the LiDAR on board and combined with the map building algorithm [26] to extract the map points of the side tracks and flippers, obtaining the original point cloud information; then the point cloud is inflated and resampled to obtain the set of terrain points inflated, which is denoted as the set *T*(the red dotted line in Figure 5, where the points are denoted as p∈T.

### 3.2. Model Calculation

The calculation method draws on the literature  [27], the posture of a robot in terrain is determined by its own configuration and the contact points with the terrain. Assuming that the ground reference frame is [G], the pose prediction problem can be described in the following form. There are the following definitions:

- *T*: The set of points in the terrain.
- $x_{R}$: The x-axis coordinate of the chassis center in [G]
- $\theta_{f}$: The angle of the robot’s front flipper, the counterclockwise is positive.
- $\theta_{r}$: The angle of the robot’s rear flipper, the clockwise is positive.

Solving for robots in a static steady state:

- $y_{R}$: Coordinate on the y-axis of the center of the chassis in [G].
- ϕ: The pitch angle of the robot in [G], positive when counterclockwise.

Figure 5 illustrates the specific meaning of the above parameters. Solving the pose is to find the terrain points, that is, the contact points, from the set of points *T* that satisfy the constraints and then determine the pose parameters. Here, the pose of the robot is calculated in the ‘steady state’, based on which two constraints can be imposed:

- Condition 1: The existence of more than one point of contact with the terrain on each side of the front and back of the robot’s center of mass is an essential condition for stability;
- Condition 2: The pose of the robot, determined by the contact points, must ensure that every point of the robot lies above all points of the terrain within its reachable range—a fundamental physical constraint that prevents collisions with the ground.

**Figure 6:**
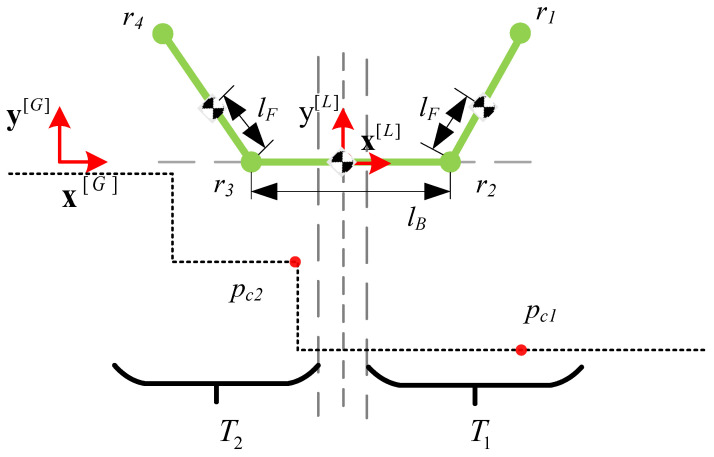
Segmented model of a horizontally placed robot in local coordinate system.

Establish a local coordinate system [*L*] with a y-axis parallel to the direction of gravity and an x-axis in the direction of robot orientation, using the position to be predicted ($x_{R}$, $y_{R}$) in [*G*] as the origin. By making the center of the robot chassis coincide with the origin and the chassis parallel to the x axis, the model shown in Figure 6 can be obtained. The model can be described by segmented straight lines for each component:

(1) $$K=\left\{\begin{array}{cc} l_{r_{2}r_{1}} & \mathrm{front}\;\mathrm{flipper}\;r_{2}r_{1}\\ l_{r_{3}r_{2}} & \mathrm{chassis}\;r_{3}r_{2}\\ l_{r_{4}r_{3}} & \mathrm{rear}\;\mathrm{flipper}\;r_{4}r_{3} \end{array}\right.$$

The terrain point–robot contact can be represented as a point on a segmented linear model. Specifically, the robot *K* is first rotated around the origin at the pitch angle ϕ in the frame [L], then transformed to $K^{\prime }$ by translating *h* along the y axis and is in contact with the terrain points $p_{c1}\left(x_{c1},y_{c1}\right)\in T_{1}$ and $p_{c2}\left(x_{c2},y_{c2}\right)\in T_{2}$, as shown in Figure 6.

To satisfy constraint 1, the terrain points must be located on the front and back sides of the robot’s center of mass. The geometric relationship between a point and a line can be expressed as the product of the dot of the homogeneous coordinate vector of the line and the homogeneous coordinate vector of the point being zero.

**Figure 7:**
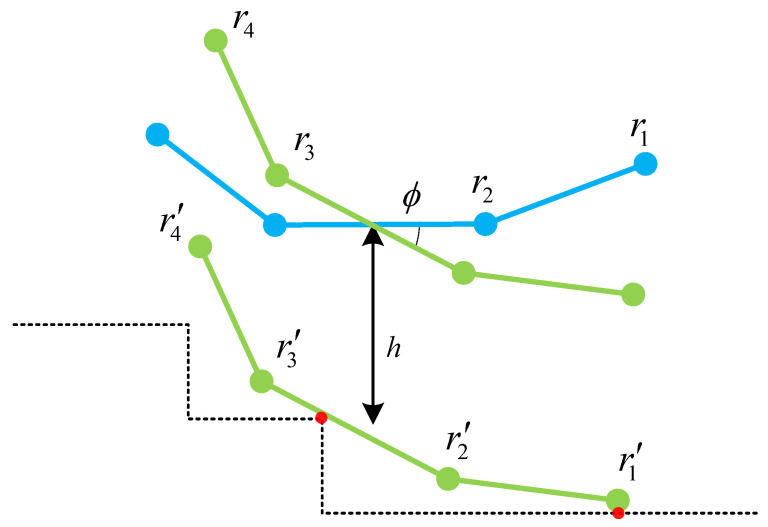
Schematic diagram of rotating and then translating the robot model.

$p_{c1}$ and $p_{c2}$ may be located on the flipper or on the chassis, the geometric relationship is as follows:

(2) $$\left\{\begin{array}{cc} l_{r_{1}^{\prime }r_{2}^{\prime }}^{T}\cdot p_{c1}=0, & p_{c1}\;\mathrm{on}\;\mathrm{the}\;\mathrm{front}\;\mathrm{flipper},x_{c1}\in \left(x_{r_{2}^{\prime }},x_{r_{1}^{\prime }}\right]\\ l_{r_{4}^{\prime }r_{3}^{\prime }}^{T}\cdot p_{c1}=0, & p_{c1}\;\mathrm{on}\;\mathrm{the}\;\mathrm{chassis},x_{c1}\in \left(x_{\mathrm{center}},x_{r_{2}^{\prime }}\right] \end{array}\right.$$

(3) $$\left\{\begin{array}{cc} l_{r_{3}^{\prime }r_{2}^{\prime }}^{T}\cdot p_{c2}=0, & p_{c2}\;\mathrm{on}\;\mathrm{the}\;\mathrm{chassis},\;x_{c2}\in \left(x_{r_{3}^{\prime }},x_{\mathrm{center}}\right]\\ l_{r_{4}^{\prime }r_{3}^{\prime }}^{T}\cdot p_{c2}=0, & p_{c2}\;\mathrm{on}\;\mathrm{the}\;\mathrm{rear}\;\mathrm{flipper},\;x_{c2}\in \left(x_{r_{4}^{\prime }},x_{r_{3}^{\prime }}\right] \end{array}\right.$$

where: $l_{{r_{i+1}}^{\prime }{r_{i}}^{\prime }}\left(i=1,2,3\right)$ is the line that passes through points and in the transformed robot model *K*, $p_{cj}$(*j* = 1, 2) is the contact point expressed as a homogeneous coordinate vector, as shown in Equation (4).

(4) $$p_{cj}=\left[\begin{array}{c} x_{cj}\\ y_{cj}\\ 1 \end{array}\right],\;l_{r_{i+1}^{\prime }r_{i}^{\prime }}=\left[\begin{array}{c} y_{r_{i+1}^{\prime }}-y_{r_{i}^{\prime }}\\ x_{r_{i+1}^{\prime }}-x_{r_{i}^{\prime }}\\ x_{r_{i+1}^{\prime }}y_{r_{i}^{\prime }}-x_{r_{i}^{\prime }}y_{r_{i+1}^{\prime }} \end{array}\right]$$

where $\left(x_{r_{i}^{\prime }},y_{r_{i}^{\prime }}\right)$ are the coordinates of $r_{i}^{\prime }$, which are obtained by rotating and translating $r_{i}$ in the initial state in Figure 7, as in Equation (5).

(5) $$\left[\begin{array}{c} x_{r_{i}^{\prime }}\left(h,\phi \right)\\ y_{r_{i}^{\prime }}\left(h,\phi \right) \end{array}\right]=\left[\begin{array}{cc} \cos\phi & -\sin\phi \\ \sin\phi & \cos\phi \end{array}\right]\left[\begin{array}{c} x_{r_{i}}\\ y_{r_{i}} \end{array}\right]+\left[\begin{array}{c} 0\\ h \end{array}\right]$$

In Constraint 1, the contact points also need to satisfy the requirement that they are distributed in front of and behind the center of mass, that is, the range of x-axis coordinates of the contact points in Equations (2) and (3) should be far away from the center of mass. The coordinates ($x_{center},y_{center}$) of the center of mass of the robot model in [*L*] can be expressed as Equation (6):

(6) $$\left\{\begin{array}{c} x_{center}=\frac{1}{m_{B}+2m_{F}}\left[m_{F}l_{F}\left(\cos\theta_{f}-\cos\theta_{r}\right)+m_{B}x_{b}\right],\\ y_{center}=\frac{1}{m_{B}+2m_{F}}\left[m_{F}l_{F}\left(\sin\theta_{f}+\sin\theta_{r}\right)+m_{B}y_{b}\right]. \end{array}\right.$$

where ($x_{b}$, $y_{b}$) are the coordinates of the chassis center of mass, $l_{0}$ is the distance from the flipper center of mass to the flipper rotational axis, and $m_{B}$ and $m_{F}$ are the masses of the robot’s unilateral chassis as well as the individual flippers. Assuming that the chassis center of mass coincides with the center of the chassis shape, that is ($x_{b}$, $y_{b}$) = (0, 0), we have Equation (7):

(7) $${x_{center}}^{2}+{y_{center}}^{2}=2\left[1-\cos\left(\theta_{f}+\theta_{r}\right)\right]{\left(\frac{m_{F}l_{F}}{m_{B}+2m_{F}}\right)}^{2}\leq {\left(\frac{2m_{F}l_{F}}{m_{B}+2m_{F}}\right)}^{2}$$

In other words, the center point ($x_{center},y_{center}$) varies within a circle with a radius of $\frac{2m_{F}l_{F}}{m_{B}+m_{F}}$, with the center of gravity of the vehicle chassis as the origin.

(8) $$x_{center}\in \left[-\frac{2m_{F}l_{F}}{m_{B}+m_{F}},\;\frac{2m_{F}l_{F}}{m_{B}+m_{F}}\right]$$

We can initially divide all the terrain points under [*L*] into two subsets before and after $T_{1}$ and $T_{2}$ with the upper and lower boundaries of the $x_{center}$ as boundaries, and set the candidate contact points to be located in them, i.e., $p_{c1}$($x_{c1}$,$y_{c1}$) $\in T_{1},\;p_{c2}$($x_{c2}$,$y_{c2})\;\in T_{2}$ respectively (Figure 6). The position of a point relative to a line can be expressed as the dot product of and *p*, positive or negative. Therefore, this condition can be transformed into Equation (8):

(9) $$\left\{\begin{array}{cc} l_{r_{2}^{\prime }r_{1}^{\prime }}^{T}\cdot p\leq 0, & x_{p}\in \left(x_{r_{2}^{\prime }},x_{r_{1}^{\prime }}\right]\\ l_{r_{3}^{\prime }r_{2}^{\prime }}^{T}\cdot p\leq 0, & x_{p}\in \left[x_{r_{3}^{\prime }},x_{r_{2}^{\prime }}\right],\;p\left(x_{p},y_{p}\right)\in T\\ l_{r_{4}^{\prime }r_{3}^{\prime }}^{T}\cdot p\leq 0, & x_{p}\in \left[x_{r_{4}^{\prime }},x_{r_{3}^{\prime }}\right) \end{array}\right.$$

The (*h*, ϕ) corresponding to the candidate contact points $p_{c1}$ and $p_{c2}$ that finally satisfy all the conditions is the predicted pose of the robot at the current position. Associative Equations (2)–(5) and (8) can be solved. A feasible solution may not always exist for certain terrain configurations, and the invalid solution corresponds to the robot configuration and terrain location, i.e., the non-drivable point, which will be avoided in the path planning.

When the total number of terrain points is limited, the system of equational equations consisting of (2) and (3) can be utilized to traverse to solve the analytical solutions of robot quickly poses corresponding to all the candidate contact point pairs ($p_{c1}$, $p_{c2}$) in the local terrain point set. Possibilities that satisfy all the constraints can be obtained by filtering them according to the interval range condition and inequality condition. Based on this idea, a tracked robot pose prediction method that can be applied to complex terrain is designed. Firstly, a pair of candidate contact points ($p_{c1}$, $p_{c2}$) are selected from the terrain point sets $T_{1}$ and $T_{2}$, and since the contact points may be located either on the flipper or the chassis, (2) and (3) can be composed into a system of equations to be solved by proposing one equation each according to the following cases:

- $p_{c1}$ is on the front flipper, and $p_{c2}$ is on the rear flipper;
- $p_{c1}$ is on the front flipper and $p_{c2}$ is on the chassis;
- $p_{c1}$ is on the chassis, and $p_{c2}$ is on the rear flipper;
- $p_{c1}$ is on the chassis, and $p_{c2}$ is on the chassis.

The basic form of these systems of equations is the same, and the second case is used next as an example, while the other three instances will just replace the corresponding parameters. Substituting Equations (4) and (5) into the system of equations consisting of the first line of Equation (2) and the first line of Equation (3), so that *t* = tan(ϕ/2), then sin $\phi =2t/(1+t^{2})$ and cos $\phi =\left(1-t^{2}\right)/\left(1+t^{2}\right)$. The system of equations about h and t is obtained by organizing as in Equation (9):

(10) $$\left\{\begin{array}{c} \left(A_{1}-X_{1}h\right)\;t^{2}+\left(B_{1}-2Y_{1}h\right)\;t+\left(C_{1}+X_{1}h\right)=0\\ \left(A_{2}-X_{2}h\right)\;t^{2}+\left(B_{2}-2Y_{2}h\right)\;t+\left(C_{2}+X_{2}h\right)=0 \end{array}\right.$$

The coefficients $A_{i},B_{i},C_{i},X_{i},Y_{i}\left(i=1,2\right)$ are constants related only to the original coordinates of the robot model ($x_{ri},y_{ri}$) and the coordinates of the candidate contact points ($x_{ci},y_{ci}$). Assuming that the coefficients of the highest-order terms in Equation (9) ($A_{i}-X_{i}h$) are all nonzero, expanding the two equations yields a quartic equation in *h*. Using polynomial root-finding algorithms, the solutions for *h* can be obtained. Further, using the quadratic formula and the inverse tangent function, the corresponding ϕ for *h* can be obtained. Using the same method, solutions for the other three cases can be obtained. Based on the range conditions of the x-coordinate of the contact point and the equality constraint conditions, a set of attitude parameters (*h*, ϕ) that satisfy the contact point conditions can be found among these four cases.

Based on the analytical solution method above, given a pair of candidate contact point coordinates, the attitude parameters satisfying constraint condition 1 can be calculated. Subsequently, according to Equation (8), it is determined whether constraint condition 2 is satisfied. If not satisfied, a new pair of candidate contact points is selected from $T_{1}$ and $T_{2}$, ultimately finding the single-sided robot attitude when the robot adopts the specified forward and backward swing arm angles (configuration) on the given terrain.

When the total number of terrain points in $T_{1}$ and $T_{2}$ is 30–40, the average solution time for a single pose with the specified $x_{R}$ and $\theta_{f}$ and $\theta_{r}$ is about 0.7 ms. 1000–1500 poses are predicted per second, which can satisfy the demand for real-time planning.

## 4. Flipper Motion Planning RRT* Algorithm

Flipper Motion Planning RRT* (FMP-RRT*) is proposed to solve the problem of global motion path generation for Articulated Tracked Robots in obstacle-crossing terrain. Its framework is based on Informed-RRT* [28], as Algorithm 1 shown. The following definitions are relevant:

- $x\in R^{3}$ denotes a spatial point.
- $S\in R^{4}$ denotes the state of the robot, including the height of the center of mass of the body *z*, the angle of inclination of the body ϕ, the angle of front flipper $\theta_{f}$, and the angle of rear flipper $\theta_{r}$.
- $\bar{x}\in R^{2}$ indicates a planar point projected onto the plane from space.
- $\hat{x}\in R^{3}$ denotes the location of the robot center on the terrain.
- *H* denotes a path consisting of a series of states.

Unlike traditional RRT, where each tree element represents only the position, our method defines each node as $N_{i}=\left(x_{i},s_{i},\tau_{i}\right)$, $S_{i}$ denotes the robot state,$\tau_{i}$ represents the area to which the node belongs, including the flat area, obstacle-crossing area and impassable area, its calculation is derived from Section 2. According to the above definition, given two nodes $N_{1},N_{2}$, and then $l_{12}$ denotes the Euclidean distance between them in 3D space, the cost function of the line connecting $N_{1},N_{2}$ is defined as follows:

(11) $$f\left(N_{1},N_{2}\right)=\left(1+j\omega \right)\cdot l_{12}$$

Which

(12) $$\omega =\frac{F-F_{\min}}{F_{\max}-F_{\min}}=\frac{mg\sin\alpha +\mu mg\cos\alpha -\mu mg}{mg-\mu mg}$$

(13) $$l_{12}=\sqrt{{\left(x_{2}-x_{1}\right)}^{2}+{\left(y_{2}-y_{1}\right)}^{2}+{\left(z_{2}-z_{1}\right)}^{2}}$$

(14) $$\alpha =\arcsin\left(\frac{z_{2}-z_{1}}{l_{12}}\right)$$

where *j* is the crossing penalty scale factor, ω is the crossing difficulty cost, characterized by the minimum power output of the robot crossing process and normalized, and μ is the rolling friction coefficient between the track and the ground so that the algorithm aims to minimize the path length and traversal difficulty. Pseudocode for Algorithm 1, the common subfunctions of the Informed-RRT* algorithm can be found in [28], It is briefly described as follows:

- SampleEllipsoid(): sampling is conducted in the elliptical area from the starting point to the ending point.
- RandomSample(): global random sampling.
- FindNearest(*T*, ${\bar{x}}_{rand}$): find the nearest point in the tree *T* to ${\bar{x}}_{rand}$.
- Steer($N_{nearest}$, ${\bar{x}}_{rand}$): move a fixed distance from $N_{nearest}$ to ${\bar{x}}_{rand}$ to obtain new points.
- FindNeighbors(*V*, $N_{new}$): look for the set of neighboring points of $N_{new}$ in the set of nodes *V*.
- FindParent($\Omega_{near}$, $N_{new}$): look for the parent node of $N_{new}$ in $\Omega_{near}$.
- Rewire(*T*, $\Omega_{near}$, $N_{new}$): reconnect the tree *T* to optimize the path, specifically, in the neighborhood nodes near $N_{new}$, checking whether $N_{new}$ is used as the parent node can reduce the path cost *f*($N_{new}$, $\Omega_{near}$) of these neighborhood nodes. If possible, reconnect its parent node as $N_{new}$.
- InGoalRegion($N_{new}$): determine whether $N_{new}$ is near the finish line.

Other subfunctions are described as follows.

- Pos($\bar{x}$): given 2D raster point coordinates $\bar{x}$, returns the corresponding node *N*.
- Posture Calculation ($\theta_{f}$, $\theta_{r}$, *b*, *c*): given the initial angles $\theta_{f}$ and $\theta_{r}$ of the robot’s front and rear flippers and the resolution *b* and *c* of the angle prediction, solve for $\phi_{new}$ and $y_{new}$ under different arm angle conditions to form the set of possible robot states $S_{1}$, $S_{2}$, $S_{3}$, ….
- Configuration Planning ($N_{1}$, $N_{2}$): Given two nodes, calculate the best $S_{1}$ at the $N_{2}$ node, including the best angles of the front and rear flipper $\theta_{f-best}$, $\theta_{r-best}$, the best body height $y_{best}$ and the best body angle of pitch $\phi_{best}$.

The calculation principle of the configuration planning function is as follows:
**Algorithm 1** FMP-RRT* ($N_{start}$, $N_{goal}$, *k*)  1: $V\leftarrow \{N_{start}\}$, E←∅, $\Omega_{goal}\leftarrow \emptyset$  2: T=(V,E)  3: **for **i=1 to *k* **do**  4:     **if** $H^{*}\neq \emptyset$ **then**  5:         ${\bar{x}}_{rand}\leftarrow \mathrm{SampleEllipsoid}()$  6:     **else**  7:         ${\bar{x}}_{rand}\leftarrow \mathrm{RandomSample}()$  8:     **end if**  9:     ${\bar{x}}_{nearest}\leftarrow \mathrm{FindNearest}\left(T,{\bar{x}}_{rand}\right)$10:     ${\bar{x}}_{new}\leftarrow \mathrm{Steer}\left({\bar{x}}_{nearest},{\bar{x}}_{rand}\right)$11:     $N_{new}\leftarrow \mathrm{Pos}\left({\bar{x}}_{new}\right)$12:     **if** $\tau_{new}$ =obstacle-crossing **then**13:         $\left\{s_{1},s_{2},s_{3},\ldots \right\}\leftarrow \mathrm{PostureCalculation}\left(\theta_{f},\theta_{r},b,c\right)$14:     **end if**15:     **if** $N_{new}\neq \emptyset$ and $\tau_{new}\neq \mathrm{impassable}$ and $S_{new}\neq \emptyset$ **then**16:         $\Omega_{near}\leftarrow \mathrm{FindNeighbors}\left(V,N_{\mathrm{new}}\right)$17:         **if** $\Omega_{near}\neq \emptyset$ **then**18:            $N_{parent}\leftarrow \mathrm{FindParent}\left(\Omega_{near},N_{new}\right)$19:            $V\leftarrow V\cup \{N_{new}\}$20:            $E\leftarrow E\cup \{\left(N_{parent},N_{new}\right)\}$21:            **if** $\tau_{new}=\text{obstacle-crossing}$ or $\tau_{parent}=\text{obstacle-crossing}$ **then**22:                $S_{new}\leftarrow \mathrm{ConfigurationPlanning}\left(N_{parent},N_{new}\right)$23:            **end if**24:            T←(V,E)25:            $T\leftarrow \mathrm{Rewire}(T,\Omega_{near},N_{new})$26:         **end if**27:     **end if**28:     **if** $\mathrm{InGoalRegion}(N_{new})$ **then**29:         $\Omega_{goal}\leftarrow \Omega_{goal}\cup \left\{N_{new}\right\}$30:         $H^{*}\leftarrow \mathrm{GeneratePath}\left(\Omega_{\mathrm{goal}}\right)$31:     **end if**32: **end for**33: **return** $H^{*}$

Pose planning at the two nodes according to the preset robot configuration ($\theta_{f}$, $\theta_{r}$) produces a discrete state space consisting of the path point position, front and rear flipper angles, robot pitch angle and height above the ground. The robot pose at the specified position ${\bar{x}}_{i}$ and flipper angles $\theta_{f}$, $\theta_{r}$ are defined as a discrete state $S_{{\bar{x}}_{i},\theta_{f},\theta_{b}}=\left(z\left(\bar{x},\theta_{f},\theta_{b}\right),\phi \left(\bar{x},\theta_{f},\theta_{b}\right)\right)$, calculated as described in Section 3, which means that the robot’s center of mass height and body pitch angle are determined by the position the robot is at $\theta_{f}$, $\theta_{r}$, and the front and rear flipper angles are calculated. The angles that the front and rear flippers change between adjacent path points are indicated as $\Delta \theta_{f},\Delta \theta_{r}$, in the range [−bc,…−b,0,b…,bc] b∈Z; where *b* is the discretized resolution of the flipper angle and *c* is the number of unit resolutions that the flipper joint angle can change at most whenever the robot advances one path point. Based on the experience of human operators, a cost function *g* is designed to evaluate the cost of taking an action($\Delta \theta_{f},\Delta \theta_{r}$) at the state $S_{{\bar{x}}_{i},\theta_{f},\theta_{b}}$:

(15) $$g\left({\bar{x}}_{i},\theta_{f},\theta_{r},\Delta \theta_{f},\Delta \theta_{r}\right)=g_{\phi }+g_{h}+g_{s}$$

where $g_{\phi }$ is the cost of the change in pitch angle between the front and back states, $g_{z}$ is the cost of the difference between the height of the center of mass and the average height of the ground in the chassis range in the current state, and $g_{s}$ is the cost of the stability of the pitch direction in the current state. The definitions of the three are as follows.

(16) $$g_{\phi }=\omega_{\phi }{∥\phi_{{\bar{x}}_{i+1},\theta_{f}+\Delta \theta_{f},\theta_{r}+\Delta \theta_{r}}-\phi_{{\bar{x}}_{i},\theta_{f},\theta_{r}}∥}^{2}$$

(17) $$g_{h}=\omega_{h}{∥h_{\mathrm{mean}}-z_{{\bar{x}}_{i+1},\theta_{f}+\Delta \theta_{f},\theta_{r}+\Delta \theta_{r}}∥}^{2}$$

(18) $$g_{s}=\omega_{s}{∥\phi_{{\bar{x}}_{i+1},\theta_{f}+\Delta \theta_{f},\theta_{r}+\Delta \theta_{r}}∥}^{2}$$

$\omega_{\phi },\omega_{h}$ and $\omega_{s}$ represent the cost weighting coefficients for their respective functions, which determine the proportion of each cost in the total cost. These coefficients are tuned on the basis of empirical data. In this study, our goal is to achieve a balanced cost distribution among the three, thus setting each coefficient to 1/3. $h_{mean}$ represents the average height of the local shape in the current terrain. After obtaining the robot states in various configurations at each point of the path and the generation values when taking multiple actions, the dynamic programming algorithm is used to find the optimal state transfer path in the state space of the robot connecting the start state and the goal state, so that the cumulative cost value is minimized.

(19) $$g_{\mathrm{all}}\left(S_{\bar{x},\theta_{f},\theta_{b}}\right)=\min\;\left\{g_{\mathrm{all}}\left(S_{\bar{x}-1,\theta_{f}-\Delta \theta_{f},\theta_{b}-\Delta \theta_{b}}\right)+g\left(S_{\bar{x},\theta_{f}+\Delta \theta_{f},\theta_{r}+\Delta \theta_{r}}\right)\right\}$$

The specific steps of the dynamic programming algorithm are as follows:

- Step 1: Specify the robot’s starting node $N_{parent}$ and the desired target node $N_{new}$. At this point, the robot state set at the desired target node has already been calculated by the preceding ’PostureCalculation function’ (line 13 of the pseudocode algorithm).
- Step 2: Identify the optimal state within the target node’s robot state set that minimizes the cost function gall. This state becomes the new node’s $s_{new}$.
- Step 3: Upon encountering the next desired target node, calculate the cumulative cost of all previous transition states. Update the optimal state for all nodes, along with the optimal front/rear flippers angles $\theta_{f-best}$, $\theta_{r-best}$, chassis height $z_{best-new}$ and chassis pitch angle $\phi_{best-new}$.

The FMP-RRT* algorithm iteratively samples the configuration space and refines the path via rewiring to asymptotically converge to the optimal solution.Specifically, the FMP-RRT* algorithm samples and unfolds in a 2D method to obtain a new planar point $\bar{x}$. Then $\bar{x}$ mapping to the spatial point, a new $N_{new}$ node is generated, and subsequently, the region attributes of this node are judged; if it belongs to the obstacle-crossing region, the prediction of flipper angle is carried out, and all the possible poses are computed given the initial angle of the flipper and the angular resolution; next, the FMP-RRT* algorithm connects the $N_{new}$ to the tree, and if the validity of the $N_{new}$ is verified by collision checking, and the flipper angle exists a feasible solution, then the father node of the new node is found, and the optimal flipper angle change from the parent node to the child node is computed, and then the path reconnection is performed so that the path cost is minimized; the FMP-RRT* algorithm repeats the above operation until it iterates to the specified maximal iteration tree and returns the optimal solution H*. The FMP -RRT* algorithm has the following advantages in addition to inheriting the fast search and convergence speed of the Informed-RRT* algorithm: Firstly, this algorithm plans the obstacle-crossing maneuvers and paths simultaneously. Secondly, this algorithm does not add more computation while increasing the planning of the flipper action and only predicts the flipper action in the obstacle-crossing region during the unfolding of the random sampling tree, which reduces the computation of terrain analysis, avoids useless analysis, and accelerates the response speed of the algorithm.

## 5. Experiments and Results

**Figure 8:**
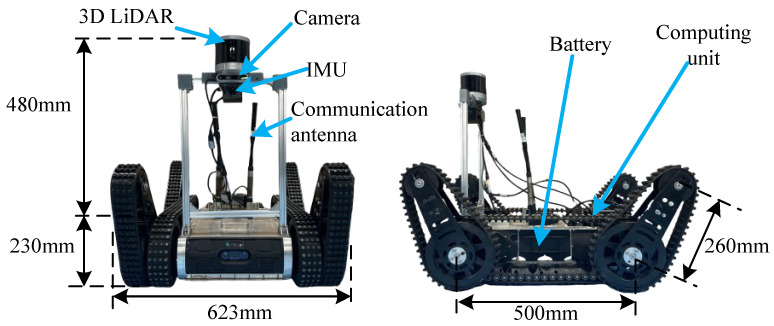
Articulated tracked robot: Climber.

As shown in Figure 8, the experiment utilizes the articulated tracked robot Climber. The LiDAR sensor is the Robosence Helios 32, while the IMU sensor is the WIT 9073. Together, they serve as mapping and localization sensors. The computing unit is an NVIDIA Orin, featuring an ARM Cortex-A78 CPU and 64GB of memory to run all algorithms. The camera is a Microsoft D435i, used to capture video information in front of the robot (for manual operation). The communication antenna facilitates data transmission between the robot and a remote host computer. The battery supplies power to the robot.We utilize the DLIO algorithm [29] to combine LiDAR scans and IMU data for mapping and localization. A simple PID controller is used as the motion-following controller.

### 5.1. Map Semantic Segmentation

**Figure 9:**
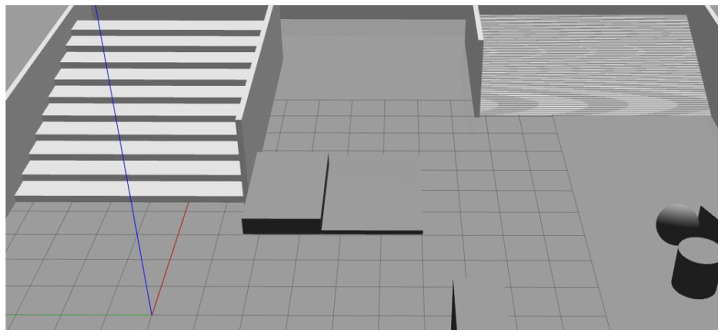
Simulation environment for making data sets.

We set up 15 environments that contain obstacle-crossing areas on the Gazebo simulation platform. The obstacle-crossing areas mainly consist of different platforms and stairs (as shown in Figure 9). We controlled the robot to move in this environment and collected a total of 2500 real-time elevation grid maps, which were mapped to images with a resolution of 224 × 224 based on height values. The data was then divided into a training dataset and a validation dataset at a ratio of 9:1. The images from the training dataset were classified into safe passage areas, obstacle-crossing areas, and impassable areas. During training, data enhancement techniques such as random flipping, random cropping, and padding were applied. The model was trained with transfer learning: In Phase 1, the feature extraction layers were frozen and only the newly added classifier head was updated. In the second phase, the training was unfrozen and the feature extraction network was included in the training. The initial learning rate is set to 0.005, the batch size to 16, and the number of epochs to 50 and 250, respectively. Focal loss is used as the loss function, and the Adam optimization algorithm is used to reduce training time and accelerate model convergence. The model’s terrain recognition accuracy is evaluated using the mean intersection over union (MIoU) and mean pixel accuracy (MPA) as metrics, while the frame rate (FPS) is used to assess the model’s inference speed.

Three common semantic segmentation models were trained on the constructed dataset. The proposed model was compared with PSPNet, UNet, and DeepLab v3+ equipped with different backbone networks. Evaluation metrics are shown in Table 1, where MIoU and MPA reached 92.44% and 94.98%, respectively. With an inference speed of 56.45 frames per second, the proposed model demonstrated the highest recognition accuracy.

**Figure 10:**
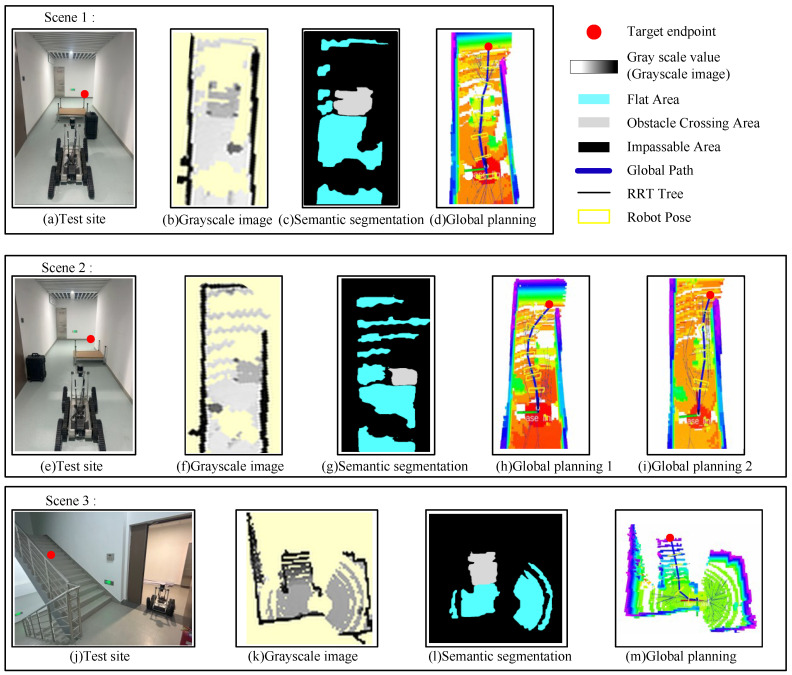
Global planning experiments for three scenarios.

The recognition performance in real-world scenarios is shown in Figure 10c,g,l. Unscanned maps and obstacles to be avoided are marked in black, flat areas are marked in blue, and obstacle-crossing areas are marked in gray. The real-time recognition time is within 80–100 ms, which meets the planning requirements. The experimental results demonstrate that this method can accurately distinguish between flat areas, obstacle-crossing areas, and obstacle-avoidance areas, facilitating motion planning.

### 5.2. Validation of Planning Algorithms

We simulated the experiments in three scenarios, setting the sample step size at 0.5 m and the Obstacle-crossing penalty scale factor *j* to 1.

Scene 1 (Figure 10a): A 22 cm high platform and a 50 cm high black box are placed in a hallway. The space on both sides of the platform is too narrow for the robot to pass, and the target point is placed in front of it. The robot autonomously plans a motion path, successfully avoiding the black box and crossing the platform (initial solution time: 265 ms, asymptotic optimization time: 782 ms).

Scene 2 (Figure 10e): Similarly to Scenario 1, but with the platform placed against one wall, leaving sufficient passage on the left, while the black box is near the opposite wall. The robot initially plans a path, avoiding both obstacles (initial solution time: 223 ms, asymptotic optimization time: 563 ms). When the obstacle-crossing penalty factor j is adjusted to 0.5, the robot instead plans a path crossing the platform, demonstrating the influence of the penalty factor in the cost function.

Scene 3 (Figure 10g): The robot starts at the entrance of the staircase with the target point on the stairs. It successfully generates a 3D path to climb the stairs (initial solution time: 326 ms, asymptotic optimization time: 628 ms).

Experiments across these three scenarios demonstrate that the proposed planning framework can adapt to diverse obstacle-crossing scenarios, enabling obstacle-crossing planning for classic obstacles such as stairs and steps within an acceptable timeframe.

### 5.3. Movement Quality Analysis

We quantitatively compared autonomous planning with professional operator teleoperation in Scenarios using three key metrics:

- Time: total movement time.
- Max ϕ: the maximum absolute pitch angle, the larger it is, the more likely the robot will fall.
- Rot.Ang: the cumulative absolute pitch angle of the front and rear flippers.

The robot’s maximum speed is limited to 0.2 m/s, with a maximum acceleration of 0.3 $m/s^{2}$ and a fixed flipper angular velocity of $25^{\circ }/s$. We performed five operations and calculated the average metrics. The same initial and target positions were set for each scene. The results are shown in the following Table 2.

Analysis of the four motion sequences in three scenarios reveals that the autonomous movement method requires consistently less time than the manual operation method in all scenarios. This is because climbing elevated platforms and stairs requires coordinated forward-backward flippers. Improper control can cause the robot to sway or even tip over during ascent. The additional complexity of the control often leads operators to reduce speed, thus increasing movement time. In contrast, autonomous movement offers a significant advantage, achieving faster speeds when autonomously planning obstacle-crossing actions. This shows that the proposed planning method is more efficient during task execution. During movement, autonomous and manual operations exhibit similar movements, resulting in comparable Max ϕ values. In Scenario 2’s obstacle avoidance, both values are zero because no obstacle-crossing action occurs, eliminating the need for flipper movement. In other scenarios, the sum of Max ϕ and the rotation angle for autonomous motion is consistently lower than that for manual operation. This is because when approaching the boundary between obstacles and flat ground—where flipper angle control is required—autonomous motion avoids redundant swinging present in manual operation, indicating superior stability. Autonomous motion also eliminates the repeated flipper angle adjustments required in manual operation, resulting in a smaller Max ϕ. This indicates that autonomous motion offers superior smoothness and reduced energy consumption.

## 6. Conclusions

This paper introduces an autonomous planning framework tailored for articulated tracked robots navigating complex terrains with obstacle-crossing maneuvers. The framework aims to achieve efficient autonomous navigation through semantic segmentation of the map and collaborative planning. Specifically, a method based on the MB-DeepLab V3+ network is introduced for semantic segmentation, accurately classifying terrain into flat, obstacle-crossing, and impassable areas. A simplified robot-terrain contact model is constructed to enable rapid calculation of the robot’s pose at map sampling points through contact point traversal, thus avoiding complex geometric calculations and enhancing planning efficiency while ensuring stability in complex terrain. Additionally, the FMP-RRT* global path planning algorithm is proposed, which incorporates the smoothness cost of flipper motions into the search process to achieve collaborative planning of obstacle-crossing paths and flipper motions, substantially improving the efficiency and stability of path planning. Experiments demonstrate that the MB-DeepLab V3+ semantic segmentation algorithm achieves high efficiency and high precision segmentation. The entire framework outperforms manual control in terms of travel time and stability, while also reducing flipper oscillations and enhancing movement smoothness. Future efforts will concentrate on optimizing the real-time performance and robustness of the algorithm to tackle increasingly complex and dynamic environments.

## Figures and Tables

**Table 1:** Comparison of different semantic segmentation algorithms.

Algorithm	Backbone	MIOU/%	MPA/%	FPS (Frame/s)
PSPNet	Resnet50	82.37	92.35	16.67
	Mobilenet v2	83.51	91.88	**65.89**
UNet	Resnet50	79.22	91.43	10.38
	Vgg	81.23	91.68	6.35
DeepLab V3+	Xception	78.98	91.26	10.10
	Mobilenet V2	84.87	92.24	25.13
	Mobilenet V3-Large	91.31	93.77	29.57
	Mobilenet V3-Small	88.22	93.45	57.33
**MB-DeepLab V3+**		**92.44**	**93.98**	56.45

**Table 2:** Performance Comparison Between Manual Operation and Autonomous Movement Across Multiple Scenarios.

Scenario	Method	Time (s)	Max ϕ (^∘^)	Rot. Ang (^∘^)
1	Manual operation	40.48	37.28	478.36
	Autonomous movement	30.23	30.56	386.56
2-crossing	Manual operation	42.42	37.62	435.98
	Autonomous movement	31.44	30.88	390.23
2-avoiding	Manual operation	28.32	0.00	0.00
	Autonomous movement	25.14	0.00	0.00
3	Manual operation	30.23	31.23	268.21
	Autonomous movement	26.56	29.32	196.36

## Data Availability

The dataset that supports the central findings of this study is directly available in this article. Additional data can be requested from the corresponding author.

## Abbreviations

The following abbreviations are used in this manuscript:

ATRs	Articulated Tracked Robot
RRT	Rapidly-exploring Random Tree
FMP-RRT*	Flipper Motion Planning-Rapidly Exploring Random Tree
BAM	Boundary Aware Module
